# The accuracy of anal self- and companion exams among sexual minority men and transgender women: a prospective analysis

**DOI:** 10.1016/j.lana.2024.100704

**Published:** 2024-02-29

**Authors:** Alan G. Nyitray, Timothy L. McAuliffe, Cameron Liebert, Michael D. Swartz, Ashish A. Deshmukh, Elizabeth Y. Chiao, Lou Weaver, Ellen Almirol, Jared Kerman, John A. Schneider, J. Michael Wilkerson, Lu-Yu Hwang, Derek Smith, Aniruddha Hazra, The Prevent Anal Cancer Palpation Study Team

**Affiliations:** a Clinical Cancer Center, Medical College of Wisconsin, Milwaukee, WI, USA; b Center for AIDS Intervention Research, Medical College of Wisconsin, Milwaukee, WI, USA; c School of Medicine and Public Health, University of Wisconsin–Madison, USA; d Department of Biostatistics and Data Science, The University of Texas Health Science Center at Houston School of Public Health, Houston, TX, USA; e Department of Public Health Sciences, Medical University of South Carolina, Charleston, SC, USA; f Cancer Control Program, Hollings Cancer Center, Medical University of South Carolina, Charleston, SC, USA; g MD Anderson Cancer Center, Houston, TX, USA; h University of Chicago, Section of Infectious Diseases and Global Health, Chicago, USA; i Department of Health Promotion and Behavioral Sciences, The University of Texas Health Science Center at Houston School of Public Health, Houston, TX, USA; j Department of Epidemiology, Human Genetics, and Environmental Sciences, The University of Texas Health Science Center at Houston School of Public Health, Houston, TX, USA; k The Crofoot Research Center, Houston, TX, USA

**Keywords:** Anus neoplasms, Early detection of cancer, Digital anal rectal examination, HIV, Anal canal, Homosexuality, Male

## Abstract

**Background:**

Squamous cell carcinoma of the anus (SCCA) annual incidence among sexual minority men with and without HIV is 85/100,000 and 19/100,000 persons, respectively, which is significantly higher than the overall incidence (2/100,000). Incidence may also be higher in transgender women. Since SCCA tumours average ≥30 mm at diagnosis, we assessed the accuracy of individuals to self-detect smaller anal abnormalities.

**Methods:**

Using convenience sampling, the study enrolled sexual minority men and transgender women, aged 25–81 years, in Chicago, Illinois and Houston, Texas, USA, during 2020–2022. Individuals were taught the anal self-examination and anal companion examination (ASE/ACE). Then, a clinician performed a digital anal rectal examination (DARE) before participants conducted the ASE or ACE. The sensitivity, specificity and concordance of the ASE/ACE to detect an abnormality were measured along with factors associated with ASE/ACE and DARE concordance.

**Findings:**

Among 714 enrolled individuals, the median age was 40 years (interquartile range, 32–54), 36.8% (259/703) were living with HIV, and 47.0% (334/710), 23.4% (166/710), and 23.0% (163/710) were non-Hispanic white, non-Hispanic Black, and Hispanic, respectively. A total of 94.1% (671/713) identified as cisgendered men, and 5.9% (42/713) as gender minorities. A total of 658 participants completed an ASE and 28 couples (56 partners) completed an ACE. Clinicians detected abnormalities in 34.3% (245/714) of individuals. The abnormalities were a median of 3 mm in diameter. Sensitivity and specificity of the ASE/ACE was 59.6% (95% CI 53.5–65.7%) and 80.2% (95% CI 76.6–83.8%), respectively. Overall concordance was 0.73 (95% CI 0.70–0.76) between ASE/ACE and DARE and increased with increasing anal canal lesion size (p = 0.02). Concordance was lower when participants were older and received ASE/ACE training from a lay person rather than a clinician.

**Interpretation:**

Sexual minority men/transgender women may self-detect SCCA when malignant lesions are much smaller than the current mean dimension at presentation of ≥30 mm.

**Funding:**

10.13039/100000054National Cancer Institute.

**Research in context**

**Evidence before this study**

While squamous cell carcinoma of the anus (SCCA) incidence is substantially elevated in people with HIV, there are currently no consensus recommendations on how to screen for it, nor is there widespread technological infrastructure for a prevailing method, high-resolution anoscopy. In the absence of screening programs, the size of SCCA tumours at diagnosis average ≥30 mm in greatest dimension. We searched PubMed for articles between January 1, 2000 and June 15, 2023, using the search terms ‘anus neoplasm’ and ‘self-examination’. We found no studies assessing the accuracy of self-examinations to detect anal masses other than our prior feasibility study.

**Added value of this study**

In this sample of 714 individuals, robust measures of sensitivity and specificity were derived for lay examinations that sought to detect an abnormality of the anal canal or perianus. In addition, the study established that as lesion size increases, so does concordance between clinician’s exam and the lay exam.

**Implications of all the available evidence**

When discussing anal cancer screening with patients, clinicians may advise that self- and partner examination of the anal region may result in detection of invasive tumours when they are smaller and easier to treat, although it is not a substitute for a clinician’s examination.

## Introduction

Squamous cell carcinoma of the anus (SCCA) disproportionately affects people with HIV, especially sexual minority men, among whom annual incidence is 85/100,000 persons. Incidence is also increased among HIV-negative sexual minority men (19/100,000 persons),^1^ and possibly transgender individuals.^2^

While there are no consensus screening recommendations for SCCA, an annual DARE is currently recommended by the Centres for Disease Control and Prevention,^3^ in part, because most anal cancers present with a palpable or visible tumour.^4^ The recommendation applies to all people with HIV and HIV-negative sexual minority men with a history of receptive anal intercourse.^3^ SCCA lesions are large at presentation, with a mean size of 32 mm in a 2023 Australian study^5^ and a median of 30 mm in a 2020 Chinese study.^6^ Tumour size is a strong predictor of survival and recurrence.^7^ At diagnosis, 45.5% and 44.8% of cases are at a localized or regional/distant stage, respectively.^8^ SCCA tumours ≤20 mm in greatest dimension are considered stage 1 cancer unless there is nodal involvement which indicates stage 2. Tumours >20 mm and ≤50 mm are stage 2, and those >50 mm are stage 3 or higher.^9^ In addition, superficially invasive SCCA tumours, a condition more amenable to local excision and conservative surgical management, have a horizontal spread of ≤7 mm in diameter.^10^

Due to SCCA tumour size, it seems possible that individuals could self-detect tumours through palpation or visualization of lesions.^11^ If so, self-detection could downstage SCCA especially if it stimulates care-seeking for those less likely to attend screening, e.g., due to poor HRA infrastructure, embarrassment, lack of insurance, or medical mistrust.^12^^,^^13^ Previously, we established the feasibility of teaching anal self-examinations and anal companion examinations (ASE/ACE) in addition to their preliminary accuracy estimates.^14^ In the current study, our primary objective which was to compare individuals’ ASE or ACE result with a clinician gold-standard DARE at the baseline visit to establish robust sensitivity and specificity for these exams and to determine factors associated with concordance between lay and clinician exams.

## Methods

### Recruitment and protocol

The Prevent Anal Cancer (PAC) Palpation Study (NCT04090060), based in Chicago, Illinois and Houston, Texas, USA, used a defined protocol in each city with study procedures approved by human protections committees at the Medical College of Wisconsin, the University of Chicago, The University of Texas MD Anderson Cancer Center, and the University of Texas Health Sciences Center. All individuals provided written consent for the study. The study followed STARD guidelines for reporting of research on diagnostic accuracy.^15^

Study recruitment occurred primarily through social media (i.e., Scruff, Growlr, Facebook, and Instagram), friends, in-clinic advertising, and flyers placed in lesbian, gay, bisexual, and transgender (LGBT)-friendly businesses from January 2020 to December 2022. Individuals were included if they were cisgender or transgender sexual minority men or transgender women, aged >25 years, and reported sex with men (cis or trans) in the last five years (or identified with a minority sexual orientation). Individuals were excluded if they self-reported an unresolved medical diagnosis of anal condyloma, haemorrhoids, or SCCA or if they reported a DARE in the prior 3 months. People speaking English were included in Chicago, while English and Spanish speakers were included in Houston. Recruitment was stratified to enrol single individuals and partners in couples.

Interested individuals used a URL or QR code to access the online eligibility survey. Prior to the COVID-19 pandemic eligible individuals attended a face-to-face consenting session followed by ASE/ACE instruction and assessment of exam accuracy. The study was suspended due to COVID-19 pandemic stay-at-home orders on March 14, 2020. After study resumption on July 30, 2020, in Chicago and October 1, 2020, in Houston, each individual signed a consent virtually,^16^ indicated if they would participate as an individual or a couple, and then were scheduled for an in-person appointment for ASE/ACE instruction and assessment of exam accuracy.

Appointments in Chicago occurred at a community centre serving primarily Black sexual minority men/transgender women (97.0%, 359/370) and a hospital clinic (3.0%, 11/370). In Houston, appointments occurred at private clinic serving LGBT communities (80.2%, 276/344) and a public HIV clinic (19.8%, 68/344). In the original protocol all appointments were to occur at the Houston public HIV clinic; however, the COVID-19 pandemic required having most visits at the private LGBT clinic. The venue change, in turn, affected recruitment: In Chicago, a majority were recruited through social media (64.3%, 236/367) while in Houston, a plurality were recruited through clinics (42.7%, 147/344) (Supplementary Table S1).

Physicians and advanced practice providers (APP) were trained in DARE technique and recording of findings. They practiced on mannequins and were observed by senior physicians (JAS and EYC) when conducting the initial DAREs on participants. In Chicago, a physician (AH) conducted DARE on 301 of 370 participants (81.4%) with the remainder conducted by an APP. In Houston, only APPs conducted DARE with 72.9% of DAREs (272/344) conducted by one APP (DS). The principal investigator and study staff tested HCPs’ assessment of lesion size on occasion by having the HCP palpate a latex lesion and guess its size without looking at the lesion. The HCP then received the results and was tested again using a differently sized lesion.

The ASE/ACE training for study participants included education about anal anatomy, anal cancer, and conducting an ASE/ACE. To assess non-clinician’s ability to provide the education, staff (for example, project coordinators) trained 628 of 714 participants (88%). Individual and couple-specific illustrated instructions taught participants to use a mirror or take a selfie to view the perianus (for ASE participants only), and to palpate the entire 360° of the anal canal. Participants were taught to insert their index finger as deep as the second knuckle (proximal interphalangeal joint) since the full length of the anal canal (3–5 cm) is shorter than a cisgender male index finger.^17^ Participants practiced the exam on two mannequins (Kyoto Kagaku Co., Kyoto, Japan), one with no anal canal lesion, and the other a palpable 7 mm lesion. The training emphasized that the goal was only to detect if an abnormality was present (yes or no), regardless of type (e.g., wart, haemorrhoid, fissure, or tumour); thus, we assessed ASE and ACE for multiple anal conditions with the logic that if a mass or induration, regardless of aetiology, can be palpated or seen, then malignant tumours may be recognized too. Couples conducted the exam on each other after asking permission of their partner. Participants were told that if they found an abnormality, it was very unlikely to be cancer because other conditions like haemorrhoids and condyloma were much more common. Prostate and distal rectal palpation were not taught.^18^ The training lasted on average 14 min.

The HCP then collected an anal swab and performed a DARE according to published guidelines.^19^ The HCP used the DARE as a teachable moment, e.g., “I’m feeling all 360° of your anal canal.” The HCP withheld providing DARE results until later in the visit. In addition to recording presence of an abnormality, HCPs also recorded location, size, and other characteristics for up to four abnormalities per person.

Participants were left alone in the exam room with gloves, mirror, and hand-cleaning supplies to complete the ASE or ACE and record presence of any abnormality. Participants completed the exam in an average of 4 min. Finally, the individual or couple completed a post-exam survey, received their DARE results, any needed clinical referrals, and were paid $50.

### Statistical methods

The accuracy of the ASE and ACE was determined by estimating the sensitivity, specificity, and concordance of the lay examination to identify an abnormality using the gold standard of the clinician’s DARE. The sensitivity of DARE has been estimated at 90%.^20^ Sensitivity of the ASE/ACE was stratified by the following variables and 95% confidence intervals (CI) were examined by strata for overlap: type of exam (ASE vs. ACE), anatomic site (anal canal vs. perianal), HIV, waist size, obesity, lesions needing referral (vs. those needing no referral), and self-reported dexterity-related comorbidities (i.e., arthritis, carpal tunnel syndrome, cerebral palsy, diabetes, fibromyalgia, chronic lower back pain, motor neuron diseases, movement disorders, multiple sclerosis, obesity, spina bifida, spinal cord injury, or stroke). All hypothesis tests were two-sided with a 0.05 alpha. We also estimated positive predictive value, and negative predictive value. For couples, each partner performed digital insertion on the other partner; thus, couples provided two participant results while the clinician produced two associated DARE results.

Factors associated with participant and HCP exam agreement, i.e., concordance, were assessed with 95% CI. For individuals, concordance was coded as one if the result of both HCP and participant was the same and zero if clinician and participant disagreed. For partners, concordance was coded as one if both clinician and the partner performing digital insertion agreed on the results for the other partner and zero if the performing partner and clinician disagreed. Concordance trends by lesion size were assessed with the Cochrane–Armitage test for trend. For analysis of factors associated with concordance, prevalence ratios (PR), adjusted PR (aPR), and 95% CIs were calculated by bivariate and multivariable Poisson regression using a robust sandwich estimator of the variance.^21^ Variables were included in multivariable analysis if they were significant in bivariate analysis at p < 0.15. Missing data were handled by pairwise deletion. Age and city were included in all modelling as potential confounders. Waist circumference was categorized as ≤102 cm and >102 cm in accordance with prior literature identifying increased health-related problems among individuals with >102 cm waist size.^22^ Bivariate and multivariable analyses were also conducted for only those engaging in ASE (n = 658).

Given a null value for sensitivity and specificity of 50% (since we sought to rule out accuracy due only to chance), our a priori power assessment indicated that power to detect sensitivity of ≥70% among 600 individuals was 0.90 and 0.81 among 100 couples using α = 0.05 and two-sided tests. Power to detect specificity ≥90% among both individuals and couples was >0.90. Based on our experience with lesions in the anal canal in the prior feasibility study, we assumed that 11% of participants would have an abnormality.^14^

Analyses were conducted using SAS 9.4 TS Level 1 M6 (SAS Institute, Cary, NC).

### Role of the funding source

The funder had no role in the design, data collection, data analysis, interpretation, or writing of the manuscript.

## Results

Of 1616 individuals found eligible (Supplementary Fig. S1), those ultimately attending a study appointment, 44.4% (717/1616), did not differ from those not attending an appointment by race or ethnicity, sex at birth, gender identity or sexual orientation. However, individuals attending an appointment were more likely to have heard of the study through a clinic, friend, or other means (p < 0.0001), to report having insurance (p = 0.02), and to reside in Houston (p < 0.0001) compared to those not attending. Of those who attended an appointment, the HCP could not complete the DARE for one person (the HCP was unable to visualize the perianal region due to hair), and three individuals did not acknowledge completing an ASE. Two of these individuals said they could not reach their anus and/or that it was too difficult to do the exam. The third person did not provide a reason. A total of 714 participants were left for analysis.

A total of 658 participants completed an ASE and 28 couples (56 partners) completed an ACE (Table 1). The median age was 40 years (range, 25–81), and 47.0% (334/710), 23.4% (166/710), and 23.0% (163/710) identified as non-Hispanic white, non-Hispanic Black, and Hispanic, respectively. A total of 94.1% (671/713) identified as a cisgendered man, and 5.9% (42/713) as a gender minority. A total of 36.8% (259/703) self-reported as a person with HIV (PWH).

**Table 1:** Characteristics of individuals conducting lay anal examinations and concordance with clinician examinations in Chicago, Illinois and Houston, Texas, USA 2020–2022.

	Enrolled	Concordance (95% CI)
Overall	714/714 (100.0%)	0.73 (0.70–0.76)
Age, years	40 (32–54)	
Age, years		
25–34	252/714 (35.3%)	0.79 (0.74–0.84)
35–44	161/714 (22.6%)	0.71 (0.64–0.78)
45–54	145/714 (20.3%)	0.72 (0.65–0.80)
55–81	156/714 (21.9%)	0.67 (0.59–0.74)
Waist circumference, cm		
≤102	473/708 (66.8%)	0.75 (0.72–0.79)
>102	235/708 (33.2%)	0.68 (0.62–0.74)
Sex at birth		
Male	703/714 (98.5%)	0.73 (0.70–0.77)
Female	11/714 (1.5%)	0.64 (0.35–0.92)
Gender identity		
Man	671/713 (94.1%)	0.72 (0.69–0.76)
Non-binary	18/713 (2.5%)	0.89 (0.74–1.00)
Transgender woman	11/713 (1.5%)	0.82 (0.59–1.00)
Transgender man	9/713 (1.3%)	0.78 (0.51–1.00)
Woman or other	4/713 (0.6%)	0.75 (0.33–1.00)
Race/ethnicity		
White, non-Hispanic	334/710 (47.0%)	0.72 (0.67–0.76)
Black, non-Hispanic	166/710 (23.4%)	0.80 (0.73–0.86)
Hispanic	163/710 (23.0%)	0.69 (0.62–0.76)
Asian, non-Hispanic	32/710 (4.5%)	0.81 (0.68–0.95)
Other, non-Hispanic^a^	15/710 (2.1%)	0.67 (0.43–0.91)
Sexual orientation		
Gay	601/713 (84.3%)	0.71 (0.68–0.75)
Bisexual	67/713 (9.4%)	0.82 (0.73–0.91)
Queer	35/713 (4.9%)	0.80 (0.67–0.93)
Heterosexual, lesbian, I don’t know or other	10/713 (1.4%)	0.90 (0.71–1.00)
HIV status, self-report		
Negative	444/703 (63.2%)	0.75 (0.71–0.79)
Positive	259/703 (36.8%)	0.70 (0.64–0.75)
Dexterity-related medical condition^b^		
No	465/692 (67.2%)	0.75 (0.71–0.79)
Yes	227/692 (32.8%)	0.68 (0.62–0.74)
Lay anal examination type		
Anal self-examination	658/714 (92.2%)	0.73 (0.70–0.76)
Anal companion examination	56/714 (7.8%)	0.73 (0.62–0.85)
Trainer type		
Clinician	86/714 (12.0%)	0.85 (0.77–0.92)
Non-clinician	628/714 (88.0%)	0.72 (0.68–0.75)
Clinician type		
Medical doctor	301/714 (42.2%)	0.78 (0.74–0.83)
Advanced practice provider	413/714 (57.8%)	0.69 (0.65–0.74)
City		
Chicago	370/714 (51.8%)	0.76 (0.72–0.80)
Houston	344/714 (48.2%)	0.70 (0.65–0.75)
Recruitment source		
Social media	304/711 (42.8%)	0.75 (0.70–0.80)
Clinics	169/711 (23.8%)	0.70 (0.62–0.76)
Friends	128/711 (18.0%)	0.73 (0.66–0.81)
Flyers/advertisement	99/711 (13.9%)	0.74 (0.65–0.82)
Other	11/711 (1.6%)	0.82 (0.59–1.00)
Lay anal examination results		
True negative	376/714 (52.7%)	n/a
True positive	146/714 (20.5%)	n/a
False negative	99/714 (13.9%)	n/a
False positive	93/714 (13.0%)	n/a
Preferred anal sex position		
Insertive	163/693 (23.5%)	0.73 (0.66–0.80)
Versatile	281/693 (40.6%)	0.74 (0.69–0.79)
Receptive	245/693 (35.4%)	0.71 (0.65–0.76)
Never had anal sex	4/693 (0.6%)	1.00 n/a
Difficulty with ASE/ACE		
Easy or very easy	640/708 (90.4%)	0.74 (0.71–0.77)
Hard or very hard	68/708 (9.6%)	0.62 (0.50–0.73)
Pain with ASE/ACE		
None	681/710 (95.9%)	0.73 (0.69–0.76)
A little	23/710 (3.2%)	0.78 (0.61–0.95)
A lot	2/710 (0.3%)	1.00 n/a
I don’t know	4/710 (0.6%)	1.00 n/a
Ever checked anus for disease		
No or I don’t know	385/713 (54.0%)	0.71 (0.67–0.76)
Yes	328/713 (46.0%)	0.75 (0.70–0.80)
Worry about getting anal cancer		
None	421/710 (59.3%)	0.74 (0.70–0.78)
A little	204/710 (28.7%)	0.70 (0.64–0.76)
Some	68/710 (9.6%)	0.82 (0.73–0.91)
Quite a lot	17/710 (2.4%)	0.47 (0.23–0.71)
Plans to do ASE/ACE in the future		
Strongly agree	508/709 (71.7%)	0.74 (0.70–0.78)
Agree	184/709 (26.0%)	0.70 (0.63–0.77)
Disagree	3/709 (0.4%)	1.00 n/a
Strongly disagree	2/709 (0.3%)	0.00 n/a
I don’t know	12/709 (1.7%)	0.77 (0.54–1.00)
Would see a doctor for a persistent anal problem		
Strongly agree	481/711 (67.7%)	0.74 (0.69–0.77)
Agree	183/711 (25.7%)	0.75 (0.69–0.82)
Disagree	12/711 (1.7%)	0.67 (0.40–0.93)
Strongly disagree	3/711 (0.4%)	0.33 (0.00–0.87)
I don’t know	32/711 (4.5%)	0.59 (0.42–0.76)
Preference for ASE/ACE or doctor-provided exam		
ASE or ACE	244/705 (34.6%)	0.75 (0.70–0.80)
Doctor-provided exam	461/705 (65.4%)	0.72 (0.68–0.76)
COVID-19 pandemic-associated enrolment date^c^		
Prior to trial suspension	20/714 (2.8%)	0.70 (0.46–0.88)
After trial resumed	694/714 (97.2%)	0.73 (0.70–0.77)

Note. Data are n (%) or median (interquartile range).

ASE/ACE, anal self-examination, or anal companion examination; n/a, not applicable.

a Other includes Native American or Alaskan Native, Hawaiian or Pacific Islander, other, and I don’t know.

b Conditions were arthritis, carpal tunnel syndrome, cerebral palsy, diabetes, fibromyalgia, chronic lower back pain, motor neuron diseases, multiple sclerosis, obesity, spina bifida, spinal cord injury, stroke, and other (neuropathy, lower back nerve compression, tremors in hand, McArdle disease, autism, scoliosis, knee pain, scapular dyskinesis, transverse myelitis, osteoporosis, thoracic outlet syndrome, cervicalgia, causalgia and herniated disc).

c Study enrollment was suspended due to COVID-19 pandemic stay-at-home orders on March 14, 2020, and then resumed on July 30, 2020, in Chicago and October 1, 2020, in Houston.

HCPs recorded ≥1 abnormality in 245/714 individuals (34.3%) with a median diameter at both anal canal and perianal region of 3 mm for the primary lesion (anal canal lesion size range, 1–8 mm; perianal lesion size range, 1–10 mm) (Table 2). The most prevalent lesion types appearing in the anal canal and perianus were described by HCPs as haemorrhoids (46.8%, 44/94) and skin folds, flaps, or tags (47.4%, 90/190), respectively. Lesion types by gender identity are in Supplementary Table S2.

**Table 2:** Primary lesion characteristics for 245 individuals in Chicago, Illinois and Houston, Texas, USA 2020–2022.

	Perianus (n = 190)^a^	Anal canal (n = 94)^a^
Clinician-detected lesions		
Lesion size, mm	3, 1–10	3, 1–8
Lesion type		
Haemorrhoid	53/190 (27.9%)	44/94 (46.8%)
Skin fold/flap/tag	90/190 (47.4%)	6/94 (6.4%)
Scar	12/190 (6.3%)	14/94 (14.9%)
Condyloma	14/190 (7.4%)	9/94 (9.6%)
Suspicious lump or thickening	2/190 (1.1%)	14/94 (14.9%)
Papule	9/190 (4.7%)	6/94 (6.4%)
Anal fissure	2/190 (1.1%)	1/94 (1.1%)
Other	5/190 (2.6%)	–
Perianal lichenification	1/190 (0.5%)	–
Perianal psoriasis	1/190 (0.5%)	–
Cyst	1/190 (0.5%)	–
HCP referred participant		
No	166/190 (87.4%)	64/94 (68.1%)
Yes	24/190 (12.6%)	30/94 (31.9%)
Lay detected lesions by ASE or ACE	76/190 (40.0%)	49/94 (52.1%)

Note. Data are median, range and n (%).

HCP, health care provider; ASE, anal self-exam; ACE, anal companion exam.

a Total number of individuals with primary lesions at either site is >245 due to lesions at both anatomic sites in some individuals.

**Fig. 1:**
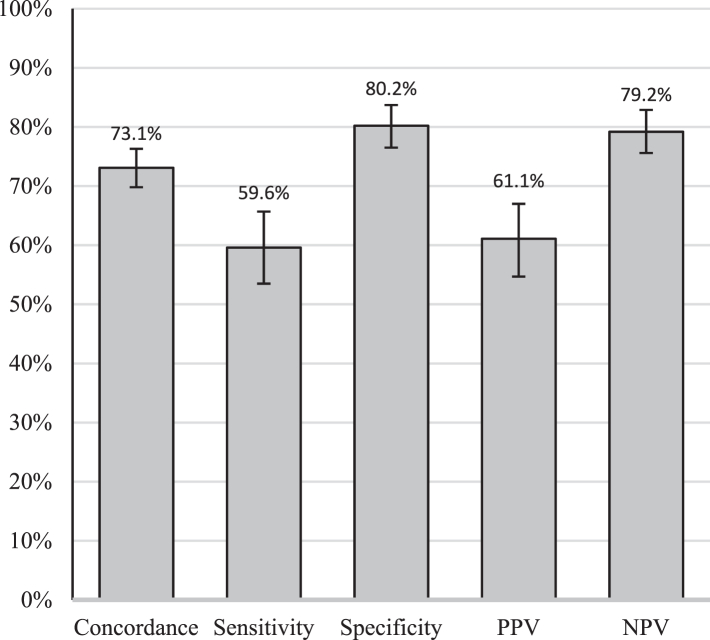
Agreement and accuracy for lay anal examinations compared with clinician examinations in Chicago, Illinois and Houston, Texas, USA 2020–2022. Abbreviations: PPV, positive predictive value; NPV, negative predictive value.

Overall sensitivity and specificity of the ASE and ACE was 59.6% (146/245) and 80.2% (376/469), respectively, while positive predictive value and negative predictive value was 61.1% (146/239) and 79.2% (376/475), respectively (Fig. 1). Accuracy differed little when assessed by exam type (Supplementary Fig. S2). When stratified by anatomic site (anal canal vs. perianus), waist size, lesions needing referral (vs. those needing no follow up), self-reported dexterity-related comorbidities, or city, no significant differences in sensitivity were observed. If a lesion prompted a clinician’s referral, sensitivity was 63.0% (29/46) (Supplementary Table S3).

The ASE/ACE had 0.73 concordance with the clinician DARE. The ASE/ACE result was a true negative for 52.7% (376/714) of all exams and true positive for an additional 20.5% (146/714) of exams (Table 1). The ASE/ACE were discordant in 27% (192/714) of exams (false negative 13.9%, 99/714; false positive 13.0%, 93/714).

**Fig. 2:**
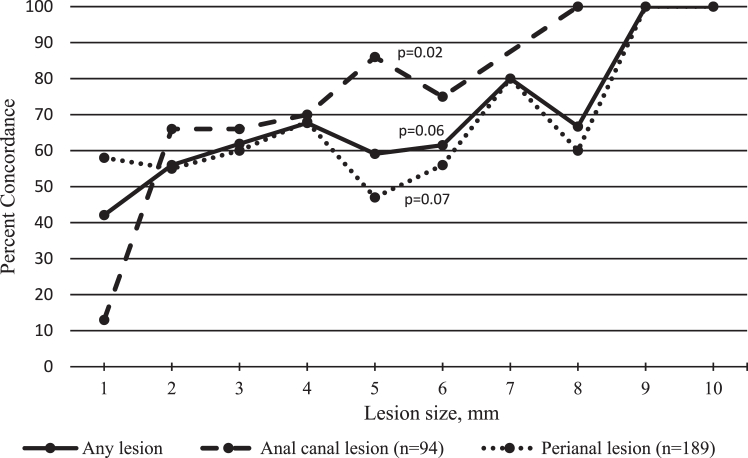
Concordance by lesion size between lay and clinician anal examinations stratified by anatomic site in Chicago, Illinois and Houston, Texas, USA 2020–2022. p value is derived from the Cochrane–Armitage test for trend. Size and number of lesions; 1 mm (n = 19), 2 mm (n = 84), 3 mm (n = 63), 4 mm (n = 31), 5 mm (n = 22), 6 mm (n = 13), 7 mm (n = 5), 8 mm (n = 6), 9 mm (n = 1), 10 mm (n = 1).

Concordance for anal canal lesions increased with increasing lesion size (p = 0.02, Fig. 2). According to a review of overlapping 95% CI, concordance was also associated with trainer type, clinician type, and amount of anal cancer worry (Table 1). Concordance was 0.85 (95% CI 0.77–0.92) when clinicians conducted the training and 0.72 (95% CI 0.68–0.75) when a non-clinician conducted the training. Concordance was 0.78 (95% CI 0.74–0.83) when a physician conducted the DARE and 0.69 when an APP conducted the DARE (95% CI 0.65–0.74). While only 17 participants reported worrying “quite a lot” about anal cancer, they had lower concordance than participants who said they worried “some” about anal cancer (0.47, 95% CI 0.23–0.71 for “quite a lot” and 0.82, 95% CI 0.73–0.91 for “some”).

After performing the ASE or ACE, 97.7% (697/709) of individuals agreed or strongly agreed they would do another in the future (Table 1). Most individuals (93.4%, 664/711) said they agreed or strongly agreed they would see a doctor for a persistent anal abnormality. A total of 34.6% (244/705) of individuals preferred the ASE/ACE exam vs. a clinician’s DARE examination.

Almost all individuals (95.9%, 681/710) said the lay exam was not painful. Of those reporting pain (n = 25), 23 reported “a little” pain and 2 individuals reported “a lot” of pain. Neither of the latter two participants required follow up.

In bivariate analysis, the oldest participants were less likely to be concordant with the clinician (PR 0.85, 95% CI 0.75–0.96 for 55–81 years old compared with 25–34 years old) as were participants with a waist size greater than 102 cm (PR 0.90, 95% CI 0.82–1.00, compared with a waist size ≤102 cm) (Table 3). A more accurate ASE/ACE was associated with Black, non-Hispanic race/ethnicity (PR 1.11, 95% CI 1.00–1.23, compared with white, non-Hispanic individuals), and a non-gay sexual orientation. For example, individuals identifying as bisexual had increased concordance (PR 1.15, 95% CI 1.02–1.30, compared with gay). The ASE/ACE were less likely to be concordant with the clinician exam when a non-clinician conducted the training vs. when a clinician conducted the training (PR 0.84, 95% CI 0.76–0.93). The ASE/ACE were also less likely to be concordant with the clinician exam when it was conducted by an APP rather than a doctor (PR 0.88, 95% CI 0.81–0.96).

**Table 3:** Factors associated with concordance between lay and clinician anal examinations in Chicago, Illinois and Houston, Texas, USA, 2020–2022, bivariate and multivariable analyses.

Characteristic	PR (95% CI)	aPR^a^ (95% CI)
Age, years		
25–34	1.0	1.0
35–44	0.91 (0.81–1.02)	0.91 (0.81–1.03)
45–54	0.92 (0.82–1.04)	0.94 (0.84–1.07)
55–81	**0.85 (0.75–0.96)**	**0.87 (0.76–0.99)**
Waist, cm		
≤102	1.0	
>102	**0.90 (0.82–1.00)**	–
Race/ethnicity		
White, non-Hispanic	1.0	
Black, non-Hispanic	**1.11 (1.00–1.23)**	–
Hispanic	0.96 (0.85–1.09)	–
Asian, non-Hispanic	1.14 (0.95–1.36)	–
Other, non-Hispanic^b^	0.93 (0.65–1.34)	–
Sexual orientation		
Gay	1.0	
Bisexual	**1.15 (1.02–1.30)**	–
Queer	1.12 (0.94–1.33)	–
Heterosexual, lesbian, don’t know or other	**1.26 (1.02–1.56)**	–
HIV status, self-report		
Negative	1.0	–
Positive	0.92 (0.84–1.02)	–
Dexterity-related medical condition		
No	1.0	–
Yes	0.91 (0.82–1.01)	–
Difficulty with ASE/ACE		
Hard or very hard	1.0	
Easy or very easy	1.20 (0.99–1.45)	–
Trainer type		
Clinician	1.0	1.0
Non-clinician	**0.84 (0.76–0.93)**	**0.87 (0.78–0.98)**
Clinician type		
Medical doctor	1.0	
Advanced practice provider	**0.88 (0.81–0.96)**	-
Worry about getting anal cancer		
None	1.0	
A little	0.95 (0.85–1.06)	–
Some	1.11 (0.99–1.26)	–
Quite a lot	0.64 (0.38–1.06)	–

Note. Confidence intervals in bold do not include unity.

PR, prevalence ratio; aPR, adjusted prevalence ratio; ASE/ACE, anal self-examination, or anal companion examination.

a Variables remaining in model are adjusted for each other and city.

b Other includes American Indian or Alaskan Native, Native Hawaiian or Pacific Islander, and other.

In multivariable analysis age was inversely associated with concordance (aPR 0.87, 95% CI 0.76–0.99 for 55–81 years compared to 25–34 years). Also, when the trainer was a non-clinician there was decreased concordance with the ASE/ASE as compared to the clinician’s training (aPR 0.87, 95% CI 0.78–0.98). When the dataset was restricted to only individuals doing the ASE, the multivariable analysis yielded comparable results except that ‘some’ worry about getting anal cancer compared to no worry was associated with increased concordance in multivariable analysis (aPR 1.14, 95% CI 1.01–1.29) (Supplementary Table S4).

## Discussion

Among people who are especially vulnerable to SCCA, HCPs recorded 245 abnormalities in the anal canal and perianus with a median diameter of 3 mm and a range of 1–10 mm. After being taught how to conduct an ASE or ACE, participants achieved 59.6% (146/245) sensitivity to detect these lesions when compared with an HCP DARE. As anal canal lesion size increased there was increasing concordance between HCPs’ DARE and participants’ ASE/ACE. These results indicate that lay individuals who complete an ASE or ACE are likely to detect SCCA at an early stage when malignant lesions are smaller than the current known median dimension at presentation of ≥30 mm.^6^

About one-third of individuals preferred the anal self- or companion exam rather than a DARE which may speak to embarrassment associated with DARE and stigma regarding anal health^12^^,^^23^; thus, the ASE/ACE may increase anal cancer screening uptake if an abnormality triggers a clinical visit. It is worth mentioning that most HCPs are not conducting DAREs as recommended.^3^^,^^24^

To our knowledge, this study is the first to assess accuracy of ASE/ACE other than our prior feasibility study among 200 sexual minority men/transgender women which also found increased concordance with larger lesions, but higher sensitivity and specificity than the current study (75% and 94%, respectively). In that study, one clinician provided all education and DAREs.^14^ The current study’s results may be closer to ASE/ACE true accuracy when considering the larger sample size, and the use of multiple clinicians and trainers including lay trainers. Since clinicians have little time to teach the ASE/ACE, our use of lay trainers is pragmatic and may reflect ASE/ACE accuracy outside of a study setting. Also, in the prior and current study, participants were more likely to palpate larger lesions. Given reports of excellent cure rates^25^ including a 100% disease-specific cure rate for anal cancer tumours ≤10 mm,^26^ self-recognition of early-stage tumours (i.e., ≤20 mm) is likely to reduce anal cancer morbidity and mortality by increasing the potential for detecting superficially invasive squamous cell carcinomas (SISCCA) and stage 1 carcinoma.

While ASE/ACE results seem to produce minimal anxiety,^27^ false-negative (13.9%, 99/714) and false-positive (13.0%, 93/714) results may lead to missed disease and unnecessary clinic visits for procedures like DARE. An annual DARE, as recommended for PWH and HIV-negative sexual minority men, could help address both false-negative and false-positive self- and companion exams; however, only 13.7% of these populations report receiving a DARE in the prior year.^24^ As with breast self-exams,^28^ it is also possible that practicing the ASE/ACE could lead to more involvement with HCPs, and thus correction of false positive results with a DARE.

Few characteristics affected concordance except for age with individuals ≥55 years having 13% lower concordance compared to individuals 25–34 years. Since anal cancer incidence increases with age, it is a limitation if older people are less likely to detect a lesion. Conversely, younger people with increased ability to detect lesions may support self-detection of common conditions like condyloma. Concordance also differed by trainer type with non-clinician educators having 13% lower concordance than clinicians, possibly due to individuals focusing more when clinicians did the training.

While exceeding our goal of enrolling participants for the self-exam, we missed our goal of enrolling 100 couples to do the companion exam. Difficulty recruiting couples was also observed in the prior study.^14^ It may be that the self-exam is preferred to engaging a partner in an exam. It also may be that stigmas associated with anal cancer, anal intercourse, etc., present barriers to couples’ communication about joining the study. Participants engaging in ACE were more likely to be married or cohabitating than single with no partner (p < 0.0001) and more likely to be white, non-Hispanic compared with Black, non-Hispanic (p = 0.04). Nevertheless, both ASE and ACE had the same concordance, 0.73, and the ACE may be beneficial for people who cannot perform the ASE, for example, due to disability.^27^

Although all clinicians received training to assess lesion diameter, clinicians did not use a scale and thus their assessed lesion size may be incorrect. However, the primary HCP in Chicago and Houston conducted 302 and 274 DAREs, respectively, which may lead to consistency in lesion sizing, but not necessarily validity. It may also be a limitation that we could only assess the lateral size of lesions and not their depth.

Treatment of high-grade squamous intraepithelial (HSIL) lesions at the perianus or anal canal will reduce the risk of invasion.^29^ It seems unlikely that HSIL can be palpated although SISCCA of the anal canal and perianus can be palpated by clinicians^4^ and thus may be palpated through self-exams too. SISCCA invites more conservative surgical management, possibly without the need for chemoradiation.^30^

We believe individuals should establish baseline anal health with an HCP-conducted DARE, at minimum. Thereafter, repeated ASE/ACE should increase an individual’s familiarity with their anus and increase recognition of subtle changes in tissue. Given high potential for SCCA recurrence,^7^ ASE/ACE could support detection of a recurring tumour along with submucosal tumours that evade detection with HRA. Finally, these exams could be used in settings that lack the option of HRA.^13^

Study strengths include the large cohort, the diversity of participants, and settings in both a clinic and a drop-in centre. Additional limitations include the COVID-19 pandemic-related protocol changes that modified consenting procedures, the clinic site in Houston, and recruitment methods.

Given high anal cancer incidence among sexual minority men, likely high incidence among transgender women, no uniform screening standard for anal cancer, and limited infrastructure for screening (in both high and low-resource settings),^13^ these results suggest that ASE/ACE may detect early-stage anal cancer when treatment is more successful.^31^ When discussing anal cancer screening with patients, clinicians may advise that self- and partner examination of the anal region may result in detection of invasive tumours when they are smaller and easier to treat, although it is not a substitute for a clinician’s DARE.^3^ The optimal frequency for performing ASE/ACE is unknown. Nevertheless, it may be a valuable community-led tool for raising awareness and for screening for anal cancer. Since 46% (328/713) of sexual minority men/transgender women reported previously using their fingers to check for anal problems (without benefit of educational instruction), and given extremely high incidence of anal cancer, future research should study ASE/ACE implementation for populations vulnerable to SCCA.

## Contributors

AGN conceptualized the study and secured funding. AGN, MDS, TLM, and EYC developed the methodology. CL, LW, AH, and JK administered the clinics and taught participants how to do the lay examinations. AH and DS conducted clinical examinations. AGN, CL, and EA managed the data. AGN, and TLM conducted the formal analysis. AGN wrote the first draft of the manuscript. AGN, TLM, CL, MDS, AAD, EYC, LW, EA, JAS, JMW, LH, DS, AH, AAD reviewed and edited manuscript drafts.

## Data sharing statement

Fully de-identified datasets and data dictionary will be shared with properly trained investigators on the study website (https://mindyourbehind.org) within 1 year of study completion after assessment of institutional policies, Medical College of Wisconsin Human Research Protections Program rules, as well as local, state, and federal laws and regulations. Further information is available from the corresponding author upon request.

## Declaration of interests

Aniruddha Hazra declared receiving consulting fees from Gilead Sciences, ViiV Healthcare, and Abbott Technologies; Ashish A. Deshmukh declared receiving consulting fees from Merck Inc., Value Analytics Lab, support to attend EUROGIN, and payment or honoraria for giving talks/conferences at the NIH and Mt. Sinai; Elizabeth Y. Chiao has a leadership or fiduciary role as Chair, solid Tumour Working Group, AIDS Malignancy Consortium; Michael D. Swartz received funding from the NIH.

## References

[1] Clifford G.M., Georges D., Shiels M.S. (2021). A meta-analysis of anal cancer incidence by risk group: toward a unified anal cancer risk scale. Int J Cancer.

[2] Silverberg M.J., Nash R., Becerra-Culqui T.A. (2017). Cohort study of cancer risk among insured transgender people. Ann Epidemiol.

[3] Centers for Disease Control & Prevention (2021). Sexually transmitted infections treatment guidelines, 2021. MMWR Recomm Rep.

[4] Berry J.M., Jay N., Cranston R.D. (2014). Progression of anal high-grade squamous intraepithelial lesions to invasive anal cancer among HIV-infected men who have sex with men. Int J Cancer.

[5] Wong J., Allwright M., Hruby G. (2023). Anal cancer: a 20-year retrospective study from Australia. ANZ J Surg.

[6] Lu Y., Wang X., Li P. (2020). Clinical characteristics and prognosis of anal squamous cell carcinoma: a retrospective audit of 144 patients from 11 cancer hospitals in southern China. BMC Cancer.

[7] Bentzen A.G., Guren M.G., Wanderas E.H. (2012). Chemoradiotherapy of anal carcinoma: survival and recurrence in an unselected national cohort. Int J Radiat Oncol Biol Phys.

[8] Surveillance Research Program NCIA (2023). https://seer.cancer.gov/statistics-network/explorer/.

[9] Janczewski L.M., Faski J., Nelson H. (2023). Survival outcomes used to generate version 9 American Joint Committee on Cancer staging system for anal cancer. CA Cancer J Clin.

[10] Darragh T.M., Colgan T.J., Cox J.T. (2012). The lower anogenital squamous terminology standardization project for HPV-associated lesions: background and consensus recommendations from the College of American pathologists and the American society for colposcopy and cervical pathology. J Low Genit Tract Dis.

[11] Ong J.J., Temple-Smith M., Chen M., Walker S., Grulich A., Fairley C.K. (2014). Exploring anal self-examination as a means of screening for anal cancer in HIV positive men who have sex with men: a qualitative study. BMC Publ Health.

[12] Newman P.A., Roberts K.J., Masongsong E., Wiley D.J. (2008). Anal cancer screening: barriers and facilitators among ethnically diverse gay, bisexual, transgender, and other men who have sex with men. J Gay Lesbian Soc Serv.

[13] Damgacioglu H., Lin Y.Y., Ortiz A.P. (2023). State variation in squamous cell carcinoma of the anus incidence and mortality, and association with HIV/AIDS and smoking in the United States. J Clin Oncol.

[14] Nyitray A.G., Hicks J.T., Hwang L.Y. (2018). A phase II clinical study to assess the feasibility of self and partner anal examinations to detect anal canal abnormalities including anal cancer. Sex Transm Infect.

[15] Bossuyt P.M., Reitsma J.B., Bruns D.E. (2015). Stard 2015: an updated list of essential items for reporting diagnostic accuracy studies. BMJ.

[16] Lawrence C., Dunkel L., McEver M. (2020). A REDCap-based model for electronic consent (eConsent): moving toward a more personalized consent. J Clin Transl Sci.

[17] Daniel W.J. (2010). Anorectal pain, bleeding and lumps. Aust Fam Physician.

[18] Koulikov D., Mamber A., Fridmans A., Abu Arafeh W., Shenfeld O.Z. (2012). Why I cannot find the prostate? Behind the subjectivity of rectal exam. ISRN Urol.

[19] Hillman R.J., Berry-Lawhorn J.M., Ong J.J. (2019). International Anal Neoplasia Society guidelines for the practice of digital anal rectal examination. J Low Genit Tract Dis.

[20] Ong J.J., Fairley C.K., Carroll S. (2016). Cost-effectiveness of screening for anal cancer using regular digital ano-rectal examinations in men who have sex with men living with HIV. J Int AIDS Soc.

[21] Spiegelman D., Hertzmark E. (2005). Easy SAS calculations for risk or prevalence ratios and differences. Am J Epidemiol.

[22] National Heart, Lung, and Blood Institute Clinical guidelines on the identification, evaluation, and treatment of overweight and obesity in adults: the evidence report. NIH publication No. 98-40831998. https://www.nhlbi.nih.gov/health-pro/guidelines/current/obesity-guidelines/e_textbook/txgd/4142.htm.

[23] Lee D.J., Consedine N.S., Spencer B.A. (2011). Barriers and facilitators to digital rectal examination screening among African-American and African-Caribbean men. Urology.

[24] Nyitray A.G., Ridolfi T.J., Nitkowski J. (2023). Digital anal rectal examination usage among individuals at increased risk for anal cancer. J Low Genit Tract Dis.

[25] Alfa-Wali M., Dalla Pria A., Nelson M., Tekkis P., Bower M. (2016). Surgical excision alone for stage T1 anal verge cancers in people living with HIV. Eur J Surg Oncol.

[26] Ortholan C., Ramaioli A., Peiffert D. (2005). Anal canal carcinoma: early-stage tumors < or =10 mm (T1 or Tis): therapeutic options and original pattern of local failure after radiotherapy. Int J Radiat Oncol Biol Phys.

[27] Flores R.A., Wilkerson J.M., Travis A. (2023). Men who have sex with men experience low anxiety and few barriers to performing anal self or companion examinations: a qualitative study of the Prevent Anal Cancer Palpation Study. Cult Health Sex.

[28] Hackshaw A.K., Paul E.A. (2003). Breast self-examination and death from breast cancer: a meta-analysis. Br J Cancer.

[29] Palefsky J.M., Lee J.Y., Jay N. (2022). Treatment of anal high-grade squamous intraepithelial lesions to prevent anal cancer. N Engl J Med.

[30] Berry J.M., Jay N., Hernandez A., Darragh T.M., Palefsky J.M. (2013). 3. Outcome of excision of early invasive squamous carcinomas of the anal canal and perianus. Sex Health.

[31] Edge S.B.A., American Joint Committee on Cancer (2010). AJCC cancer staging handbook: from the AJCC cancer staging manual.

