# Supplementary material for: The accuracy of anal self- and companion exams among sexual minority men and transgender women: a prospective analysis
Source: Lancet Reg Health Am. 2024 Feb 29;31:100704. doi: 10.1016/j.lana.2024.100704 (PMC10910307; doi:10.1016/j.lana.2024.100704)
Supplement: Supplementary Figs. S1 and S2 and Tables S1–S4 [file mmc1.pdf]

Supplementary Material

A prospective analysis of the accuracy of anal self- and companion exams among sexual minority men and transgender women: The Prevent Anal Cancer Palpation Study

Contents

Supplementary Table 1. Characteristics of individuals conducting lay anal examinations and concordance with clinician examinations by city in Chicago, Illinois and Houston, Texas, USA 2020-2022

Supplementary Figure 1. Study flow

Supplementary Table 2. Primary lesion characteristics for 245 individuals stratified by gender identity in Chicago, Illinois and Houston, Texas, USA 2020-2022

Supplementary Figure 2. Agreement and accuracy for lay anal examinations compared with clinician examinations by examination type in Chicago, Illinois and Houston, Texas, USA 2020-2022. ASE=anal self-examination, ACE=anal companion examination, PPV=positive predictive value, NPV=negative predictive value

Supplementary Table 3. Stratified sensitivity estimates for lay anal examinations compared with clinician examination in Chicago, Illinois and Houston, Texas, USA 2020-2022

Supplementary Table 4. Factors associated with concordance between anal self-examinations and clinician examinations in Chicago, Illinois and Houston, Texas, USA 2020-2022, bivariate and multivariable analyses

Supplementary Table 1. Characteristics of individuals conducting lay anal examinations and concordance with clinician examinations by city in Chicago, Illinois and Houston, Texas, USA 2020-2022

	Chicago	Concordance (95% CI)		Houston	Concordance (95% CI)	
**Overall**	370/370 (100.0%)	0.76	(0.72 to 0.81)	344/344 (100.0%)	0.70	(0.65 to 0.75)
**Age**, years	37 (30-51)			46 (35-56)		
**Age**, years						
25-34	162/370 (43.8%)	0.78	(0.72 to 0.85)	90/344 (26.2%)	0.79	(0.70 to 0.87)
35-44	92/370 (24.9%)	0.79	(0.71 to 0.88)	69/344 (20.1%)	0.61	(0.49 to 0.72)
45-54	54/370 (14.6%)	0.74	(0.62 to 0.86)	91/344 (26.5%)	0.73	(0.63 to 0.82)
55-81	62/370 (16.8%)	0.68	(0.56 to 0.79)	94/344 (27.3%)	0.66	(0.56 to 0.76)
**Waist circumference**, cm						
≤102	262/370 (70.8%)	0.81	(0.76 to 0.85)	211/338 (62.4%)	0.69	(0.63 to 0.75)
>102	108/370 (29.2%)	0.66	(0.57 to 0.75)	127/338 (37.6%)	0.71	(0.63 to 0.79)
**Sex at Birth**						
Male	368/370 (99.5%)	0.76	(0.72 to 0.81)	335/344 (97.4%)	0.70	(0.65 to 0.75)
Female	2/370 (0.5%)	0.50	(0 to 1)	9/344 (2.6%)	0.67	(0.30 to 0.93)
**Gender identity**						
Man	344/369 (93.2%)	0.76	(0.71 to 0.80)	327/344 (95.1%)	0.69	(0.64 to 0.74)
Non-binary	16/369 (4.3%)	0.88	(0.71 to 1.00)	2/344 (0.6%)	1.0	n/a
Transgender woman	5/369 (1.4%)	0.80	(0.45 to 1.00)	6/344 (1.7%)	0.83	(0.54 to 1.00)
Transgender man	1/369 (0.3%)	1.0	n/a	8/344 (2.3%)	0.75	(0.45 to 1.00)
Woman or other	3/369 (0.8%)	0.67	(0.13 to 1.00)	1/344 (0.3%)	1.0	n/a
**Race/ethnicity**						
White, non-Hispanic	149/370 (40.3%)	0.75	(0.68 to 0.82)	185/340 (54.4%)	0.70	(0.63 to 0.76)
Black, non-Hispanic	120/370 (32.4%)	0.79	(0.72 to 0.86)	46/340 (13.5%)	0.80	(0.69 to 0.92)
Hispanic	76/370 (20.5%)	0.72	(0.62 to 0.82)	87/340 (25.6%)	0.66	(0.56 to 0.76)
Asian, non-Hispanic	16/370 (4.3%)	0.88	(0.71 to 1.00)	16/340 (4.7%)	0.75	(0.54 to 0.96)
Other, non-Hispanic†	9/370 (2.4%)	0.78	(0.51 to 1.00)	6/340 (1.8%)	0.50	(0.10 to 0.90)
**Sexual orientation**						
Gay	296/370 (80.0%)	0.75	(0.70 to 0.80)	305/343 (88.9%)	0.68	(0.63 to 0.73)
Bisexual	40/370 (10.8%)	0.83	(0.71 to 0.94)	27/343 (7.9%)	0.81	(0.67 to 0.96)
Queer	28/370 (8.0%)	0.75	(0.59 to 0.91)	7/343 (2.0%)	1.0	n/a
Heterosexual, lesbian, I don't know or other	6/370 (1.6%)	0.83	(0.54 to 1.00)	4/343 (1.2%)	1.0	n/a
**HIV status**, self-report						
Negative	272/362 (65.1%)	0.78	(0.73 to 0.83)	172/341 (50.4%)	0.72	(0.65 to 0.79)
Positive	90/362 (24.9%)	0.72	(0.63 to 0.81)	169/341 (49.6%)	0.68	(0.61 to 0.75)
**Dexterity-related medical condition***						
No	258/351 (73.5%)	0.77	(0.72 to 0.82)	215/341 (63.1%)	0.72	(0.66 to 0.78)
Yes	93/351 (26.5%)	0.72	(0.62 to 0.81)	126/341 (37.0%)	0.66	(0.58 to 0.74)
**Lay anal examination type**						
Anal self-examination	352/370 (95.1%)	0.76	(0.71 to 0.80)	306/344 (89.0%)	0.70	(0.65 to 0.75)
Anal companion examination	18/370 (4.9%)	0.89	(0.74 to 1.00)	38/344 (11.1%)	0.68	(0.54 to 0.83)
**Trainer type**						
Clinician	84/370 (22.7%)	0.86	(0.78 to 0.93)	2/344 (0.6%)	0.50	(0.00 to 1.00)
Non-clinician	286/370 (77.3%)	0.73	(0.68 to 0.79)	342/344 (99.4%)	0.70	(0.65 to 0.75)
**Clinician type**						
Medical doctor	301/370 (81.4%)	0.78	(0.74 to 0.83)	0/344	0.0	n/a
Advanced practice provider	69/370 (18.7%)	0.67	(0.56 to 0.78)	344/344 (100.0%)	0.70	(0.65 to 0.75)
**Recruitment source**						
Social media	236/367 (64.3%)	0.76	(0.70 to 0.81)	68/344 (19.8%)	0.71	(0.60 to 0.81)
Clinics	22/367 (6.0%)	0.73	(0.54 to 0.91)	147/344 (42.7%)	0.69	(0.61 to 0.76)
Friends	44/367 (12.0%)	0.77	(0.65 to 0.90)	84/344 (24.4%)	0.71	(0.62 to 0.81)
Flyers/advertisement	61/367 (16.6%)	0.77	(0.67 to 0.88)	38/344 (11.1%)	0.68	(0.54 to 0.83)
Other	4/367 (1.1%)	0.75	(0.33 to 1.00)	7/344 (2.0%)	0.86	(0.60 to 1.00)
**Lay anal examination results**						
True negative	198/370 (53.5%)	n/a		178/344 (51.7%)	n/a	
True positive	84/370 (22.7%)	n/a		63/344 (18.3%)	n/a	
False negative	39/370 (10.5%)	n/a		60/344 (17.4%)	n/a	
False positive	49/370 (13.2%)	n/a		43/344 (12.5%)	n/a	
**Preferred anal sex position**						
Insertive	91/356 (25.6%)	0.77	(0.68 to 0.86)	72/337 (21.4%)	0.69	(0.59 to 0.80)
Versatile	137/356 (38.5%)	0.74	(0.67 to 0.82)	144/337 (42.7%)	0.73	(0.66 to 0.80)
Receptive	127/356 (35.7%)	0.75	(0.67 to 0.82)	118/337 (35.0%)	0.66	(0.58 to 0.75)
Never had anal sex	1/356 (0.3%)	1.0	n/a	3/337 (0.9%)	1.0	n/a
**Difficulty with ASE/ACE**						
Easy or Very Easy	330/367 (89.9%)	0.77	(0.73 to 0.82)	310/341 (90.9%)	0.71	(0.66 to 0.76)
Hard or Very Hard	37/367 (10.1%)	0.65	(0.49 to 0.80)	31/341 (9.1%)	0.58	(0.41 to 0.75)
**Pain with ASE/ACE**						
None	359/367 (97.8%)	0.76	(0.71 to 0.80)	322/343 (93.9%)	0.69	(0.64 to 0.74)
A little	6/367 (1.6%)	0.83	(0.54 to 1.00)	17/343 (5.0%)	0.76	(0.56 to 0.97)
A lot	1/367 (0.3%)	1.0	n/a	1/343 (0.3%)	1.0	n/a

I don't know	1/367	(0.3%)	1.0	n/a	3/343	(0.9%)	1.0	n/a
**Ever checked anus for disease**								
No or I don't know	196/369	(53.1%)	0.74	(0.68 to 0.80)	189/344	(54.9%)	0.69	(0.62 to 0.75)
Yes	173/369	(46.9%)	0.79	(0.73 to 0.85)	155/344	(45.1%)	0.72	(0.65 to 0.79)
**Worry about getting anal cancer**								
None	219/367	(59.7%)	0.76	(0.70 to 0.81)	202/343	(58.9%)	0.72	(0.66 to 0.78)
A little	101/367	(27.5%)	0.75	(0.67 to 0.84)	103/343	(30.0%)	0.65	(0.56 to 0.74)
Some	35/367	(9.5%)	0.89	(0.78 to 0.99)	33/343	(9.6%)	0.76	(0.61 to 0.90)
Quite a lot	12/367	(3.3%)	0.50	(0.22 to 0.78)	5/343	(1.5%)	0.60	(0.22 to 1.00)
**Plans to do ASE/ACE in the future**								
Strongly agree	257/366	(70.2%)	0.78	(0.73 to 0.83)	251/343	(73.2%)	0.70	(0.64 to 0.76)
Agree	99/366	(27.1%)	0.71	(0.62 to 0.80)	85/343	(24.8%)	0.71	(0.61 to 0.80)
Disagree	1/366	(0.3%)	1.0	n/a	2/343	(0.6%)	1.0	n/a
Strongly Disagree	2/366	(0.6%)	0.0	n/a	0/343		0.0	n/a
I don't know	7/366	(1.9%)	1.0	n/a	5/343	(1.5%)	0.40	(0.00 to 0.83)
**Would see a doctor for a persistent anal problem**								
Strongly agree	245/368	(66.6%)	0.76	(0.71 to 0.82)	236/343	(68.8%)	0.70	(0.65 to 0.76)
Agree	96/368	(26.1%)	0.82	(0.75 to 0.90)	87/343	(25.4%)	0.69	(0.59 to 0.79)
Disagree	5/368	(1.4%)	0.60	(0.17 to 1.00)	7/343	(2.0%)	0.71	(0.38 to 1.00)
Strongly Disagree	3/368	(0.8%)	0.33	(0.00 to 0.87)	0/343		0.0	n/a
I don't know	19/368	(5.2%)	0.53	(0.30 to 0.75)	13/343	(3.8%)	0.69	(0.44 to 0.94)
**Preference for ASE/ACE or doctor-provided exam§**								
ASE or ACE	121/364	(33.2%)	0.80	(0.73 to 0.87)	123/341	(36.1%)	0.70	(0.62 to 0.78)
Doctor-provided exam	243/364	(66.8%)	0.74	(0.68 to 0.79)	218/341	(63.9%)	0.71	(0.65 to 0.77)

Data are n (%) or median (interquartile range). † Other includes Native American or Alaskan Native, Hawaiian or Pacific Islander, other, and I don't know. * Conditions were arthritis, carpal tunnel syndrome, cerebral palsy, diabetes, fibromyalgia, chronic lower back pain, motor neuron diseases, multiple sclerosis, obesity, spina bifida, spinal cord injury, stroke, and other (neuropathy, lower back nerve compression, tremors in hand, McArdle disease, autism, scoliosis, knee pain, scapular dyskinesis, transverse myelitis, osteoporosis, thoracic outlet syndrome, cervicalgia, causalgia and herniated disc). Abbreviations: ASE/ACE, anal self-examination, or anal companion examination; n/a, not applicable.

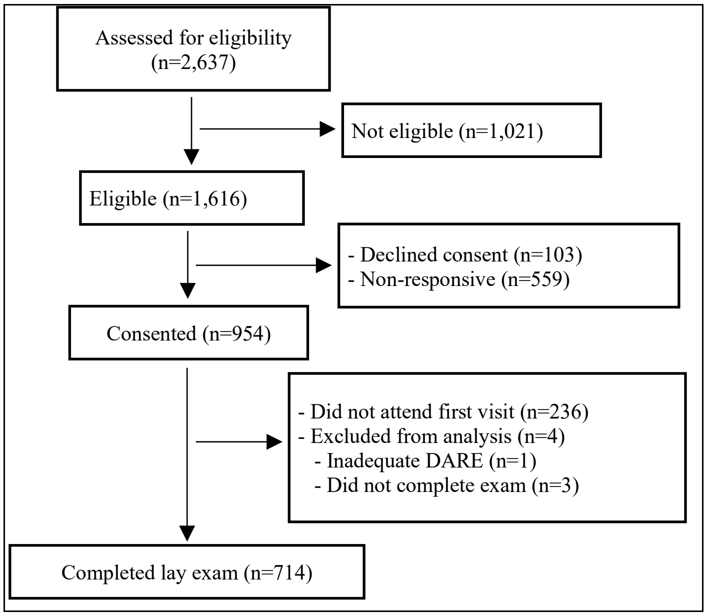

Supplementary Figure 1. Study flow.

Supplementary Table 2. Primary lesion characteristics for 245 individuals by gender identity in Chicago, Illinois and Houston, Texas, USA 2020-2022

	**Gender identity (# of participants)***							
	**Man (n=236)**		**Non-binary (n=2)**		**Transgender woman (n=3)**		**Transgender Man (n=4)**	
	**Anatomic site (# of participants)** [†]							
	**Perianus (n=183)**	**Anal canal (n=90)**	**Perianus (n=1)**	**Anal canal (n=2)**	**Perianus (n=3)**	**Anal canal (n=1)**	**Perianus (n=3)**	**Anal canal (n=1)**
**Clinician-detected lesions**								
**Lesion size**, mm, IQR	3, 2-4	3, 2-3	6, 6-6	3, 3-3	3, 1-10	3, n/a	4, 3-6	2, n/a
**Lesion type**								
Haemorrhoids	53/183 (29.0%)	43/90 (47.8%)						1/1 (100.0%)
Skin fold/flaps/tags	87/183 (47.5%)	6/90 (6.7%)					3/3 (100.0%)	
Scar	11/183 (6.0%)	13/90 (14.4%)		1/2 (50.0%)	1/3 (33%)			
Condyloma	12/183 (6.6%)	8/90 (8.9%)	1/1 (100.0%)	1/2 (50.0%)	1/3 (33%)			
Suspicious lump or thickening	2/183 (1.1%)	2/90 (1.1%)				1/1 (100.0%)		
Papule	9/183 (4.9%)	6/90 (6.7%)						
Anal fissure	n/a	1/90 (1.1%)						
Other	4/183 (2.2)	0/90			1/3 (33%)			
Perianal lichenification	1/183 (0.6)	n/a						
Perianal psoriasis	1/183 (0.6)	n/a						
Cyst	1/183 (0.6)	n/a						
**HCP referred participant**								
No	161/183(88.0%)	62/90 (68.9%)		1/2 (50.0%)	2/3 (66.7%)	0/1	3/3 (100.0%)	1/1 (100.0%)
Yes	22/183 (12.0%)	28/90 (31.1%)		1/2 (50.0%)	1/3 (33.3%)	1/1 (100.0%)		0

Note. Data are median, IQR and n and (%). [*] No lesions were detected in individuals identifying as Woman or Other. [†]Number of lesions at each anatomic site does not equal total number of participants with lesions due to lesions at both anatomic sites in some individuals. Abbreviations: IQR, interquartile range; n/a, not applicable; HCP, health care provider; ASE, anal self-exam; ACE, anal companion exam.

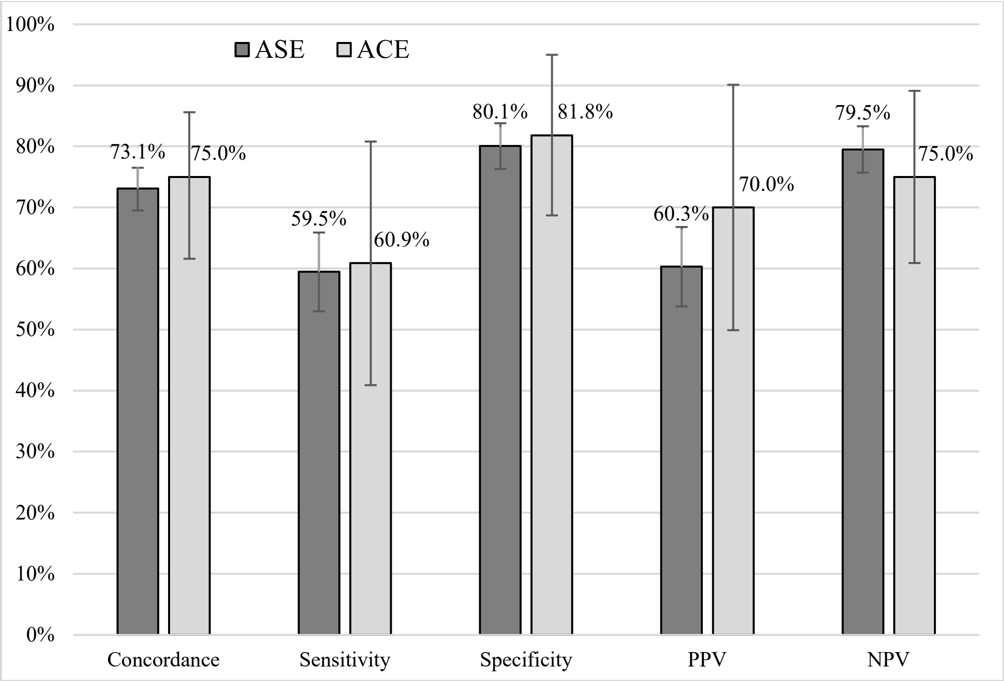

Supplementary Figure 2. Agreement and accuracy for lay anal examinations compared with clinician examinations by examination type in Chicago, Illinois and Houston, Texas, USA 2020-2022. ASE=anal self-examination, ACE=anal companion examination, PPV=positive predictive value, NPV=negative predictive value.

Supplementary Table 3. Stratified sensitivity estimates for lay anal examinations compared with clinician examinations in Chicago, Illinois and Houston, Texas, USA 2020-2022

**Stratification variable**	**Sensitivity**	**(95% CI)**
**Age**, years		
<45	64.7	(56.1-73.3)
≥45	54.8	(46.1-63.5)
**Waist circumference**, cm		
≤102	60.4	(52.9-67.9)
>102	58.0	(47.3-68.8)
**HIV status**, self-report		
Negative	62.5	(54.4-70.6)
Positive	56.2	(46.7-65.7)
**Dexterity-related medical condition**		
No	61.4	(53.7-69.2)
Yes	56.5	(45.9-67.0)
**Preferred anal sex position**		
Insertive	52.9	(39.2-66.6)
Versatile	62.4	(52.5-72.2)
Receptive	60.0	(50.2-69.9)
Never had anal sex	100.0	n/a
**Trainer type**		
Clinician	70.4	(53.2-87.6)
Non-Clinician	58.3	(51.7-64.8)
**Lesion size** (n=245)		
<5 mm	58.4	(51.5-65.3)
≥5 mm	64.6	(51.1-78.1)
**Referral for any lesion** (n=245)		
No	58.8	(52.0-65.6)
Yes	63.0	(49.1-77.0)
**Referral for anal canal lesion** (n=94)		
No	59.5	(53.0-66.1)
Yes	60.0	(42.5-77.5)
**Referral for perianal lesion** (n=190)		
No	57.9	(51.4-64.4)
Yes	75.0	(57.7-92.3)

Note. Numbers are percent.

Supplementary Table 4. Factors associated with concordance between anal self-examinations and clinician examinations in Chicago, Illinois and Houston, Texas, USA 2020-2022, bivariate and multivariable analyses

Characteristic	PR (95% CI)	aPR* (95% CI)	
**Age**, years			
25-34	1.0	1.0	
35-44	0.91 (0.81-1.03)	0.92	(0.81-1.04)
45-54	0.92 (0.81-1.04	0.94	(0.83-1.06)
55-81	**0.83 (0.73-0.95)**	**0.85**	**(0.74-0.97)**
**Waist**, cm			
≤ 102	1.0		
> 102	0.92 (0.83-1.02)	-	
**Sexual orientation**			
Gay	1.0		
Bisexual	**1.15 (1.01-1.31)**	-	
Queer	1.11 (0.93-1.33)	-	
Heterosexual, lesbian, don't know or other	**1.26 (1.02-1.56)**	-	
**HIV status**, self-report			
Negative	1.0	-	
Positive	0.92 (0.83-1.02)	-	
**Difficulty with ASE/ACE**			
Hard or Very Hard	1.0		
Easy or Very Easy	1.20 (0.99-1.46)	-	
**Trainer type**			
Clinician	1.0	1.0	
Non-clinician	**0.84 (0.76-0.93)**	**0.86**	**(0.77-0.97)**
**Clinician type**			
Medical doctor	1.0		
Advanced practice provider	**0.89 (0.81-0.98)**	**-**	
**Worried about getting anal cancer**			
None	1.0	1.0	
A little	0.95 (0.85-1.06)	0.97	(0.86-1.08)
Some	**1.13 (1.00-1.28)**	**1.14**	**(1.01-1.29)**
Quite a lot	0.63 (0.37-1.09)	0.63	(0.37-1.06)

Note. Confidence intervals in bold do not include unity. * Variables remaining in model are adjusted for each other and city. Abbreviations: PR, prevalence ratio; aPR, adjusted prevalence ratio; ASE/ACE, anal self-examination, or anal companion examination.
